# p120 Modulates LPS-Induced NF-****κ****B Activation Partially through RhoA in Bronchial Epithelial Cells

**DOI:** 10.1155/2014/932340

**Published:** 2014-06-03

**Authors:** Lingzhi Qin, Shenghui Qin, Yanli Zhang, Chao Zhang, Heng Ma, Naping Li, Liwei Liu, Xi Wang, Renliang Wu

**Affiliations:** Key Laboratory of Pulmonary Disease of Ministry of Health of China, Institute of Pathology, Tongji Hospital, Tongji Medical College, Huazhong University of Science and Technology, Wuhan, Hubei 430030, China

## Abstract

p120-Catenin (p120) is an adherens junction protein recognized to regulate cell-cell adhesion. Emerging evidence indicates that p120 may also play an important role in inflammatory responses, and the regulatory mechanisms are still unknown. In the present study, we showed that p120 was associated with airway inflammation. p120 downregulation induced nuclear factor-**κ**B (NF-**κ**B) activation, accompanied with I**κ**B**α** degradation, p65 nuclear translocation, and increased expression of interleukin-8 (IL-8) in lipopolysaccharide (LPS)- treated C57BL mice and human bronchial epithelial cells (BECs). Moreover, we first found that p120 directly coprecipitated with RhoA in BECs. After LPS stimulation, although total RhoA and p120-bound RhoA were unchanged, RhoA activity was increased. Y27632, a ROCK inhibitor, could partially inhibit nuclear translocation of p65. Overexpression of p120 inactivated RhoA and NF-**κ**B in BECs, whereas p120 loss significantly increased RhoA activity, p65 nuclear translocation, and IL-8 expression. Taken together, our study supports the regulatory role of p120 in airway inflammation and reveals that p120 may modulate NF-**κ**B signaling partially through RhoA.

## 1. Introduction

Airway epithelium is the first responder to diverse harmful stimuli, such as bacteria, viruses, and oxidant stress [1]. Lipopolysaccharide (LPS), a main stimulus isolated from Gram-negative bacteria, may cause airway injury, resulting in bronchial epithelial cell polarization, necrosis, and secretion of inflammatory cytokines [2, 3]. Persistent injury can cause chronic airway inflammation, which is the basis of various lung diseases, such as COPD, chronic pulmonary fibrosis, and lung cancer.

Numerous studies have implicated that the activation of nuclear factor-*κ*B (NF-*κ*B) signaling pathway is involved in the cell growth, proliferation, apoptosis, inflammatory gene transcription, and so forth [4–6]. It is the core of inflammatory reactions, having close connection with LPS-induced airway epithelium inflammation [2].

p120-Catenin (p120) belongs to the Armadillo protein superfamily and is originally identified as a substrate for oncogenic Src family tyrosine kinase [7]. It is best known for binding directly to the cytoplasmic domain of cadherin or VE-cadherin and contributing to regulation of cell-cell adhesion [8–10]. However, recent studies revealed that conditional deletion of p120 in the oral cavity, esophagus, and forestomach in mice results in inflammation, desmoplasia, and even invasive squamous cell cancer [11]. There is growing evidence that p120 is associated with skin, salivary glands, airway inflammation, and inflammatory bowel diseases through activating the NF-*κ*B signaling pathway [12–15]. But how p120 modulates NF-*κ*B activation in inflammatory responses is still unclear.

Regarding their connection, the activity of RhoA is the possible intermediaries in this signaling pathway. RhoA, the founder member of the Rho subfamily, is the key mediator of cytoskeletal dynamics and it emerged as crucial regulator of cadherin-mediated adhesion [16, 17]. Several studies have shown a functional relationship between p120 and RhoA [18–20]. p120 may be directly responsible for regulating the activity of RhoA in adherens junctions. In NIH 3T3 cells, p120 suppresses RhoA activity in N-cadherin complexes via recruitment of p190A Rho GTPase-activating protein [21]. In the skin inflammation, p120 affects NF-*κ*B activation and immune homeostasis in part through regulation of RhoA [12, 13]. These data suggest that p120 probably modulates NF-*κ*B activation associated with RhoA. In this study, we demonstrate a novel RhoA activity dependent interaction between p120 and NF-*κ*B signaling pathway in inflammatory responses of human bronchial epithelial cells induced by LPS. Thus, p120 may modulate NF-*κ*B signaling pathway partially through RhoA in airway inflammation.

## 2. Materials and Methods

### 2.1. Animals and Lung Inflammatory Injury

Forty male C57BL/6J mice (C57 mice, 20–25 g) were used in this study. Mice were housed in cages under specific pathogen-free conditions and used in experiments at 8–12 weeks of age. Animal protocols were received by institutional review and committee approval. Mice were randomly divided into five groups (LPS 1-day, 4-day, 7-day, and 14-day groups and the control group, with 8 mice per group). Under the conditions of ether anesthesia, the LPS group mice received LPS (Sigma-Aldrich Corps., St. Louis, MO, USA, 5 mg/kg) by intratracheal injection; the control group mice received normal saline.

### 2.2. H&E Staining and Immunohistochemical Staining

Lungs were prefixed in 4% paraformaldehyde for 1 hour, then fixed in 10% neutral formalin solution overnight, followed by paraffin embedding, and cut into 4 *μ*m serial sections. Hematoxylin-eosin (H&E) staining was used for pathological observation. SP immunohistochemical staining was used to observe the expression and distribution of p120 and NF-*κ*B, with anti-p120 monoclonal antibody (1 : 200, sc-1730, Santa Cruz) and anti-NF-*κ*B monoclonal antibody (1 : 50, Cell Signal Technology).

### 2.3. Determination of Lung Vascular Permeability and Edema

After LPS administration for 0 h, 6 h, 24 h, and 48 h, the lungs were taken out and rinsed in ice saline and then weighed, dried, and reweighed. The ratio of dry-to-wet lung weight was used as an index of lung water content and edema, an indication of lung vascular permeability and inflammation.

### 2.4. Cell Culture, Cytotoxicity Assay, and LPS Treatments

16HBE 14o-cells, a simian virus 40 large T antigen transformed human bronchial epithelial cell line that retains the differentiated morphology and function of normal human bronchial epithelia [22], were a kind gift from Dr. D. Gruenert (California Pacific Medical Center, CA, USA). Cells were cultured in Dulbecco's Modified Eagle's Medium (DMEM, GibcoBRL, Paisley, UK) and supplemented with 10% heat-inactivated fetal bovine serum (FBS, GibcoBRL) and antibiotics of 1% streptomycin and penicillin, at 37°C in 5% CO_2_.

The cytotoxicity assay has been described previously [23]. When cells reached 80–90% confluence, they were rinsed two times with PBS and incubated in serum free culture medium with or without 20 *μ*g/mL LPS (according to the MTT assay, Figure 3(a)). Data represent the means of three independent experiments.

### 2.5. Plasmid and Transient Transfection

The plasmids of RcCMVp120 3A and RcCMVp120 1A were generously provided by Wang et al. [24]. Plasmids of LZRS-mp120 isoform 1A and LZRS-mp120 Δ*N* were generously provided by Anastasiadis et al. [25]. Cells in the exponential phase of growth were transiently transfected using Lipofectamine 2000 according to the manufacturer's recommendation and the method described by Tucker et al. [26] with minor modification.

### 2.6. RhoA Activity Assay

16HBE 14o-cells were prepared with ice-cold cell lysis buffer and were immediately snap-frozen in liquid nitrogen and stored at −80°C to minimize GTP hydrolysis. An aliquot was set aside for protein concentration determination using the Precision Red Advanced Protein Assay Reagent supplied with the kit. Equal protein aliquots were added to individual wells in an eight-well strip coated with an appropriate RBD, and plates were incubated on a cold orbital microplate shaker (400 rpm) at 4°C for exactly 30 min. Strips were washed and incubated with anti-RhoA primary antibody, followed by HRP-conjugated secondary antibody and then an HRP detection reagent supplied with the kit. The signal of absorbance at 490 nm was read by a microplate spectrophotometer (SpectraMax 340 Microplate Reader; molecular devices). Samples from at least three independent experiments were assayed in triplicate.

### 2.7. Enzyme-Linked Immunosorbent Assay

After C57 mice and 16HBE 14o-cells were treated with LPS at indicated time, the IL-8 concentration in tissue and cell supernatant was determined by the ELISA kit (RD, USA) according to the manufacturer's instructions. Values of optical density were measured at 450 nm. The standard curve was made by SPSS statistical software.

### 2.8. Quantitative Real-Time PCR

Total RNA was extracted with TRIzol Reagent (Invitrogen Carlsbad, CA, USA) and the concentration was measured by an ultraviolet (UV) spectrophotometer (UV-1201; Shimadzu Corporation, Kyoto, Japan). Reverse transcription (RT) was performed as described previously [27]. Real-time PCR was carried out using the SYBR-Green PCR kit (Takara, Osaka, Japan) in a Rotor-Gene 3000 machine (Corbett Life Science, Sydney, Australia). Quantitative analysis of p120 and IL-8 transcription was described previously [14]. Each reaction was performed in a 25 *μ*L volume containing 2 *μ*L of cDNA, 0.5 *μ*L of 10 *μ*M per each primer, and 12.5 *μ*L of 2 × SYBR-Green mixture. The following were the sequences of primers: p120: For: 5′-GGA CAC CCT CTG ACC CTC G-3′, Rev: 5′-GCT TGC TAA ACT TCC TCG CTC-3′, product of 122 bp. IL-8: For: 5′-ACA CTG CGC CAA CAC AGA AAT TA-3′, Rev: 5′-TTT GCT TGA AGT TTC ACT GGC ATC-3′, product of 185 bp. GAPDH: For: 5′-ACC AGC CCC AGC AAG AGC ACA AG-3′, Rev: 5′-TTC AAG GGG TCT ACA TGG CAA CTG-3′, product of 123 bp.

### 2.9. Coimmunoprecipitation and Western Blot Analysis

Coimmunoprecipitation, cytoplasmic and nuclear extracts, and Western blot analysis were performed as described previously [23].

### 2.10. RNA Interference

The human p120 small interfering RNA (p120 siRNA) oligonucleotide was a product from Santa Cruz Biotechnology, Inc. Cells were seeded on 6-well plate to 30% confluence in complete medium and transfected with p120 siRNA using Lipofectamine2000 according to the manufacturer's recommended procedure. Efficiency of knockdown by siRNA was assessed by Western blot. The nonsilencing siRNA (scramble) was used as control. After 48 h, cells were used for further analysis.

### 2.11. Statistical Analysis

Results were expressed as means ± standard deviation (SD) of experiments repeated at least three independent times. Statistical significance was determined using SPSS 19.0 software. Data were evaluated with one-way analysis of variance (ANOVA) combined with post hoc analysis (Fisher's PLSD). A value of *P* < 0.05 was considered significant.

## 3. Results

### 3.1. Acute Lung Injury Model Is Established by Intratracheal Injection of LPS

The lethal dosage of LPS is reported as 20 mg/kg [28]. LPS of 5 mg/kg was injected to establish acute lung injury model in C57BL/6J mice.

**Figure 1:**
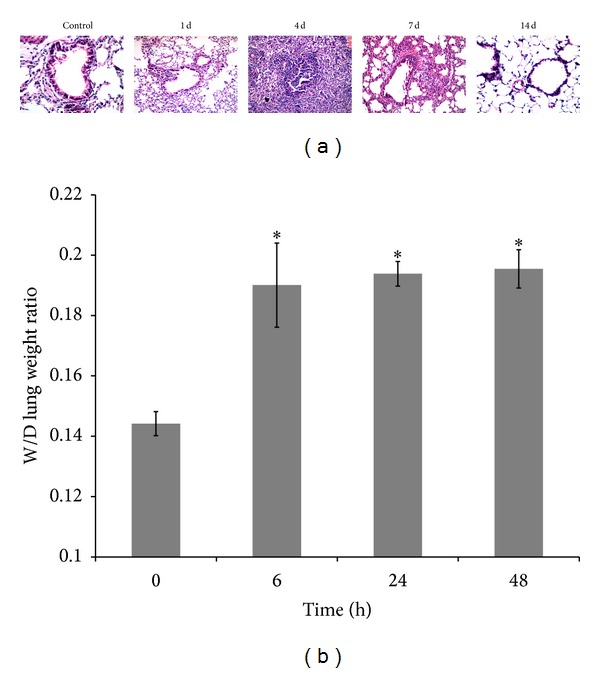
Acute lung injury was established in mice by LPS. (a) Mice were treated with LPS (5 mg/kg) through intratracheal injection. Bronchioles of 4- and 7-day experiment groups showed significant inflammatory responses. Bar = 20 *μ*m. (b) Pulmonary edema was measured by dry-to-wet lung weight ratio. Data were measured as means ± SD (*n* = 3), *P < 0.05 versus 0 h.

After LPS stimulation, morphologic changes were observed by routine H&E staining. In 4-day and 7-day groups, the airway epithelial cells underwent obvious necrosis with a large number of inflammatory cell infiltrations, alveolar septa were significantly widened with hyperemia, and perivascular spaces were also filled with inflammatory cells (Figure 1(a)). In 14-day group, the inflammatory cell infiltration was reduced, alveolar septa were no widened yet (Figure 1(a)). Meanwhile, dry-to-wet lung weight ratio showed increased lung water content and indicated the pulmonary hyperemia and edema after LPS injection. Compared with the control, the difference was statistically significant (Figure 1(b)).

### 3.2. p120 Is Downregulated and NF-*κ*B Is Activated in Lung Inflammation

**Figure 2:**
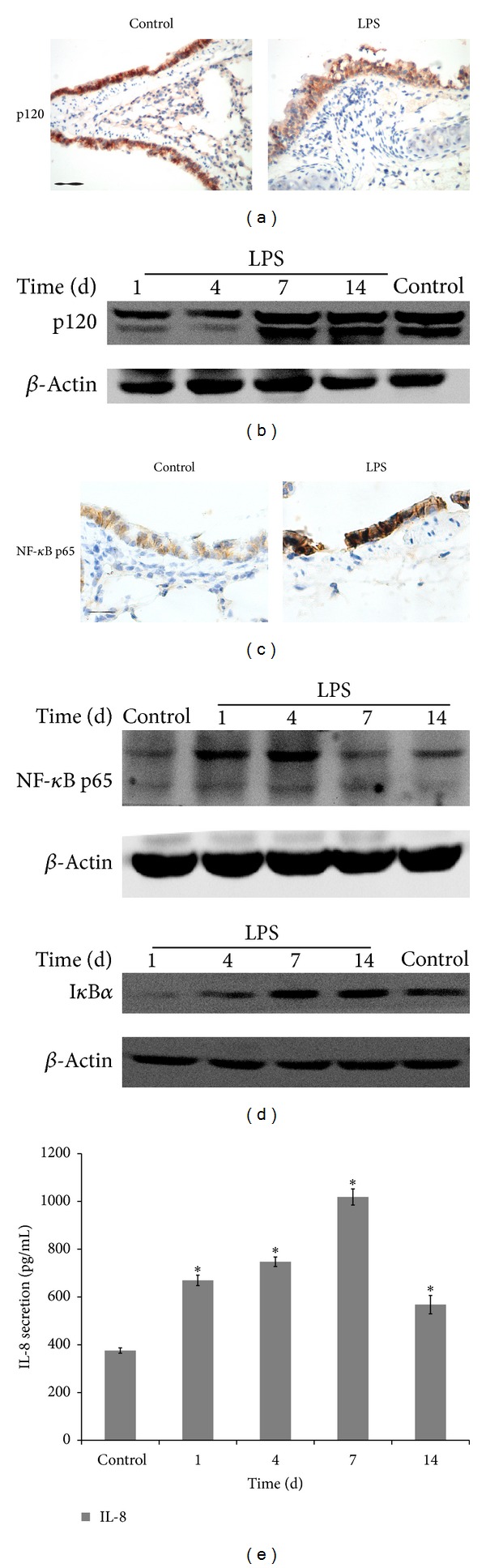
p120 downregulation activates NF-*κ*B signaling pathway in LPS-induced lung inflammation. (a) By immunohistochemical stain, the membranous expression of p120 was significantly reduced in 4-day group compared with the control; some areas showed weakened cytoplasmic p120 expression. Bar = 20 *μ*m. (b) By Western blot, p120 was downregulated in lung lysates of different groups. *β*-Actin served as internal control. (c) NF-*κ*B expression appeared nuclear distribution by immunohistochemistry (4-day group), compared with NF-*κ*B cytoplasmic expression in the control. Bar = 20 *μ*m. (d) NF-*κ*B p65 expression was increased accompanied with I*κ*B*α* degradation determined by Western blot. (e) IL-8 secretion was analyzed by ELISA, and it showed gradual increase. Data were expressed as means ± SD (*n* = 3), *P < 0.05 versus control.

Recently, researchers revealed that p120 is associated with inflammatory response. Using LPS intratracheal injection, we successfully established lung inflammation model. By immunohistochemical staining (Figure 2(a)), we found that p120 distribution changed after LPS stimulation. In control group, p120 was expressed on the lateral membrane of bronchial epithelial cells. In 4-day group, the membranous expression of p120 was significantly reduced; some areas showed weakened cytoplasmic p120 expression. By Western blot, p120 was rapidly downregulated in response to LPS challenge in the lung of 1-day and 4-day groups, isoform 1 (120 kD) and isoform 3 (100 kD) are the two major isoforms, and p120 protein expression was negatively correlated with the severity of lung inflammation (Figure 2(b)).

p120 loss may lead to NF-*κ*B activation in the skin inflammation and immune cells [11–13]. NF-*κ*B p65 subunit is responsible for the strong transcriptional activating potential of NF-*κ*B. p65 nuclear translocation and I*κ*B*α* degradation are obligatory steps in the NF-*κ*B activation [29]. By immunohistochemistry, NF-*κ*B did show apparent nuclear translocation compared with the control group in bronchial epithelial cells (Figure 2(c)). By Western blot, NF-*κ*B p65 increased significantly accompanied with the I*κ*B*α* degradation in response to LPS challenge in the lung of 1-day group and 4-day group (Figure 2(d)). Meanwhile, ELISA results indicated that NF-*κ*B target gene, proinflammatory cytokine IL-8, was gradually increased in lung tissue lysates of 1-day, 4-day, and 7-day groups and it returned to basal levels in that of 14-day group (Figure 2(e)).

### 3.3. p120 Downregulation Activates NF-*κ*B Signaling Pathway in LPS-Treated Bronchial Epithelial Cells

**Figure 3:**
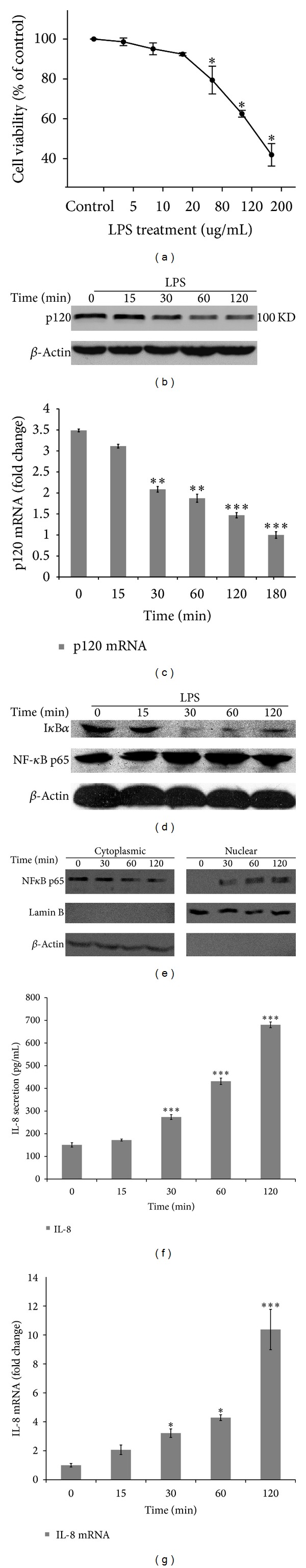
p120 downregulation activates NF-*κ*B signaling pathway in LPS-treated bronchial epithelial cells. (a) Cytotoxic effect of LPS on 16HBE 14o-cells was assessed by MTT assay. Data represent the means from three independent experiments and were analyzed by one-way ANOVA test. *P < 0.05 versuscontrol. (b) The protein expression of p120 was downregulated after being stimulated with LPS (20 *μ*g/mL). (c) Total p120 mRNA was extracted at indicated times. Fluorescent quantitative PCR was performed with SYBR-Green Mastermix PCR system. After comparing the amplifying efficiency with GAPDH, the amplification data were analyzed with the 2^−ΔΔCT^ method. The columns represented the relative amplification folds of p120 contrast to GAPDH. Data were expressed as means ± SD (*n* = 3), **P < 0.01, and ***P < 0.001 versus control. (d) NF-*κ*B p65 was increased accompanied with I*κ*B*α* degradation. (e) Nuclear translocation of p65 was detected. Equal amounts of cytoplasmic and nuclear extracts were subjected to Western blot analysis. Lamin B served as a nuclear marker, and *β*-actin served as a cytoplasmic marker. (f, g) IL-8 production was increased. (f) Supernatants were collected and assayed for IL-8 by ELISA. Data were expressed as means ± SD (*n* = 3), ***P < 0.001 versus control group (0 min). (g) IL-8 mRNA was measured by fluorescent quantitative real-time PCR. The area at 0 min was assigned as 1.0. Data were expressed as means ± SD (*n* = 3), *P < 0.05, and ***P < 0.001 versus control group (0 min).

To further confirm whether p120 was involved in airway inflammatory responses, we treated 16HBE 14o-cells with LPS* in vitro*. Cytotoxicity assay was performed after 16HBE 14o-cells were treated with different concentrations of LPS for 24 h. MTT analysis revealed a dose-dependent reduction of cell viability. Cells showed about 90% viability at 20 *μ*g/mL LPS and had only about 40% viability at 200 *μ*g/mL (Figure 3(a)). Then, we treated 16HBE 14o-cells with 20 *μ*g/mL LPS at indicated time points [14]; the results showed that both protein and mRNA levels of p120 were downregulated after LPS stimulation (Figures 3(b) and 3(c)).

Additionally, NF-*κ*B p65 was increased significantly accompanied with the degradation of I*κ*B*α* after p120 downregulation induced by LPS (Figure 3(d)). By extracting the cytoplasmic and nuclear NF-*κ*B p65, nuclear p65 was detected and markedly increased with cytoplasmic p65 decrease after LPS stimulation (Figure 3(e)). Meanwhile, the ELISA and real-time PCR results indicated that both IL-8 protein and mRNA were significantly upregulated (Figures 3(f) and 3(g)).

**Figure 4:**
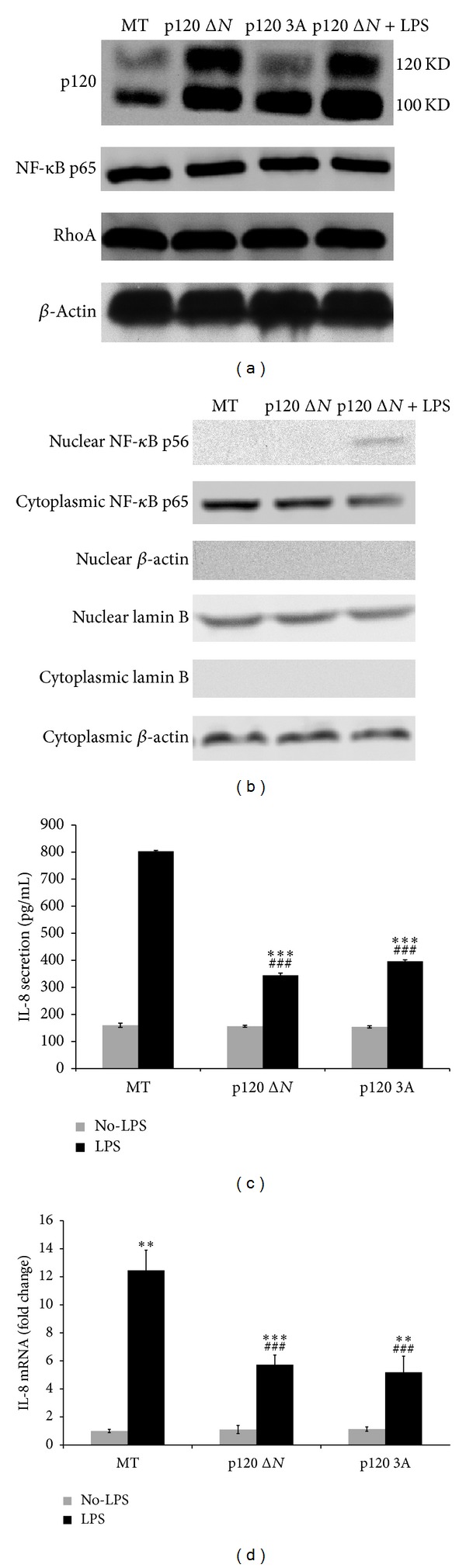
LPS-induced NF-*κ*B activation was partially inhibited by p120 transfection. (a) 16HBE 14o-cells were transiently transfected with exogenous p120 Δ*N* and p120 3A for 24 h as well as an empty vector control (mock transfection, MT). p120 Δ*N* overexpression group was additionally treated with LPS for 60 min. Both NF-*κ*B p65 and RhoA expression remained unchanged in p120 Δ*N*, p120 3A, and p120 Δ*N* + LPS groups. (b) Nuclear translocation of NF-*κ*B p65 was not found in p120 Δ*N* group, but it was detected in p120 Δ*N* + LPS group. (c, d) Overexpression of p120 reduced IL-8 production in both exogenous p120 3A and p120 Δ*N* overexpression groups compared with MT group after LPS treatment. Data were expressed as means ± SD (*n* = 3), **P < 0.01, and ***P < 0.001 versus MT + No-LPS, p120 Δ*N* + No-LPS, and p120 3A + No-LPS group, respectively. ^###^ *P* < 0.001 versus MT + LPS group.

To further elucidate the relationship between p120 isoforms and NF-*κ*B signaling pathway in bronchial epithelial cells, p120 isoform plasmids RcCMVp120 3A (p120 3A), RcCMVp120 1A, LZRS-mp120 isoform 1A, and LZRS-mp120 Δ*N* (p120 Δ*N*) were transiently transfected into 16HBE 14o-cells for 24 h, respectively, as well as an empty vector control (mock transfection, MT). The transfection efficiency was evaluated by Western blot (RcCMVp120 1A and LZRS-mp120 isoform 1A transfection efficiency< 60%; data not shown). In p120 3A, p120 Δ*N,* and p120 Δ*N* + LPS groups, overexpression of exogenous p120 has no visible effect on NF-*κ*B expression (Figure 4(a)). Although nuclear translocation of p65 was not found in p120 3A and p120 Δ*N* groups (data not shown), it was detected in p120 Δ*N* + LPS group (Figure 4(b)). After exogenous p120 3A and p120 Δ*N* transfection, the ELISA and real-time PCR showed that IL-8 production was downregulated in both groups compared with MT group after LPS treatment (Figures 4(c) and 4(d)).

**Figure 5:**
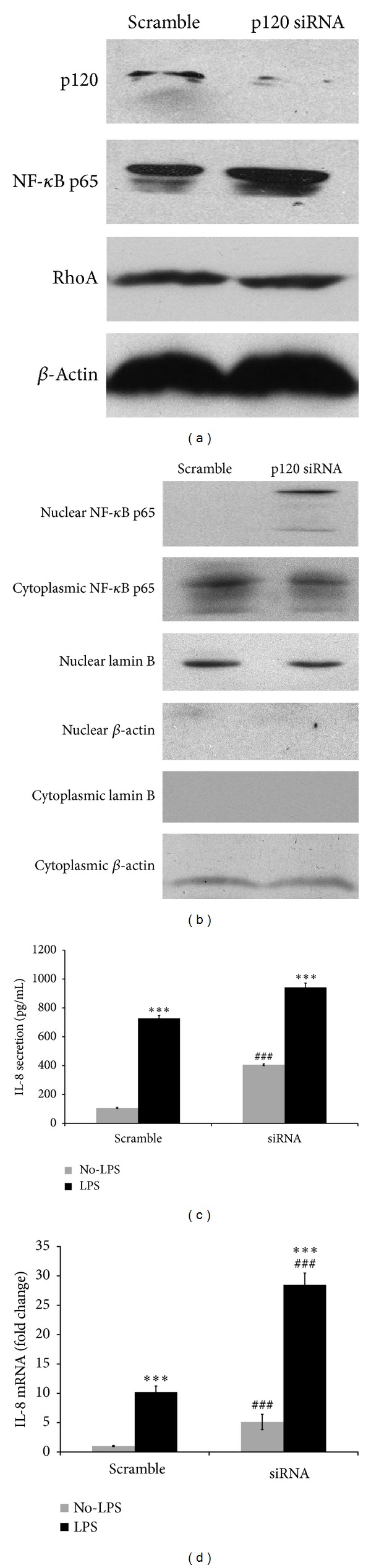
LPS-induced NF-*κ*B activation was enhanced by p120 knockdown. (a) 16HBE 14o-cells were transfected with p120 siRNA or scrambled siRNA for 48 h, p120 loss significantly increased NF-*κ*B expression, but the total RhoA remained unchanged. (b) Nuclear translocation of p65 was increased. Lamin B served as a nuclear marker, and *β*-actin served as a cytoplasmic marker. (c, d) IL-8 production was significantly upregulated both with and without LPS treatment. Data were expressed as means ± SD (*n* = 3), ***P < 0.001 versus scramble + No-LPS and siRNA + No-LPS, respectively; ^###^ *P* < 0.001 versus scramble.

Overexpression of p120 3A and p120 Δ*N* inactivates NF-*κ*B signal pathway in human bronchial epithelial cells. To validate whether p120 depletion by siRNA activates NF-*κ*B in 16HBE 14o-cells, cells were transfected with p120 siRNA or scrambled siRNA for 48 h. Western blot showed that p120 expression was significantly knocked down. NF-*κ*B p65 was increased sharply following p120 knockdown (Figure 5(a)). After p120 depletion, nuclear p65 was detected (Figure 5(b)). Meanwhile, ELISA and fluorescent quantitative real-time PCR showed a significant upregulation of IL-8 both with and without LPS stimulation (Figures 5(c) and 5(d)).

### 3.4. p120 Activates NF-*κ*B Signaling Pathway Partially through RhoA in LPS-Treated Bronchial Epithelial Cells

**Figure 6:**
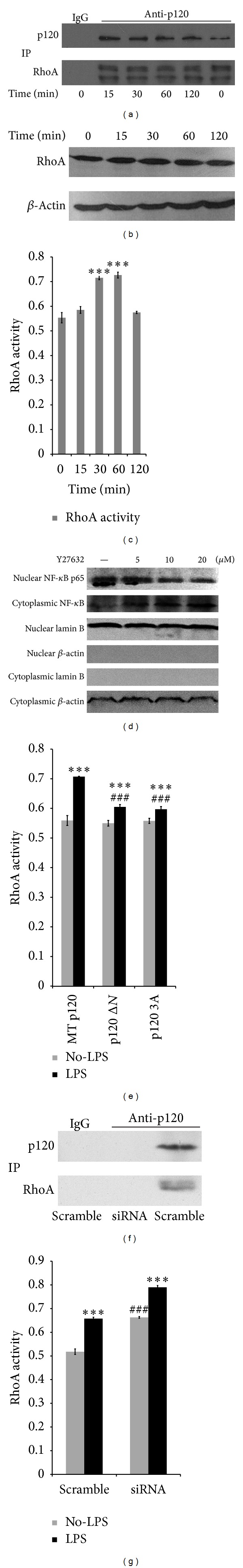
p120 activates NF-*κ*B signaling pathway partially through RhoA. (a) Coimmunoprecipitation confirmed the interaction of p120 and RhoA. The level of p120-bound RhoA was unchanged. (b) The expression of total RhoA also remained unchanged. (c) The relative level of active RhoA was increased dramatically from 30 min to 60 min by G-LISA analysis. Data were expressed as means ± SD (*n* = 6), ***P < 0.001 versus control group (0 min). (d) LPS-induced NF-*κ*B activation was inhibited by ROCK inhibitor Y27632. Cells were pretreated with Y27632 for 12 h before LPS stimulation (60 min). Nuclear translocation of NF-*κ*B p65 was partially inhibited. Lamin B served as a nuclear marker, and *β*-actin served as a cytoplasmic marker. (e) RhoA activity was decreased in both exogenous p120 3A and p120 Δ*N* overexpression groups, compared with MT group after LPS treatment. Data were expressed as means ± SD (*n* = 6), ***P < 0.001 versus MT + No-LPS, p120 Δ*N* + No-LPS, and p120 3A + No-LPS group, respectively. ^###^ *P* < 0.001 versus MT + LPS group. (f) The level of p120-bound RhoA could not be detected after p120 complete knockdown. (g) RhoA activity was sharply upregulated after p120 knockdown both with and without LPS treatment. Data were expressed as means ± SD (*n* = 6); ***P < 0.001 versus scramble + No-LPS and siRNA + No-LPS, respectively. ^###^ *P* < 0.001 versus scramble.

In skin inflammation, p120 probably modulates NF-*κ*B activation associated with RhoA [12, 13]. To study whether p120 activation of NF-*κ*B in airway inflammation is through RhoA, we investigated RhoA and its activity in 16HBE 14o-cells. Through coimmunoprecipitation, we found for the first time that RhoA could directly coprecipitate with p120 (Figure 6(a)). Although total RhoA and p120-bound RhoA were unchanged after LPS stimulation (Figures 6(a) and 6(b)), RhoA activity was increased dramatically from 30 min to 60 min (Figure 6(c)).

ROCK inhibitor Y27632 was used to further investigate their association. Before LPS stimulation, cells were pretreated with Y27632 at various concentrations for 12 h to inhibit cellular RhoA activity [30]. The results showed that nuclear translocation of p65 was partially inhibited (Figure 6(d)).

Although overexpression of exogenous p120 3A and p120 Δ*N* has no visible effect on protein levels of NF-*κ*B and RhoA (Figure 4(a)), RhoA activity was downregulated in both groups (Figure 6(e)), as well as the IL-8 production (Figures 4(c) and 4(d)), compared with MT group after LPS treatment.

Overexpression of p120 inactivates NF-*κ*B and RhoA in human bronchial epithelial cells. To further study whether p120 depletion activates NF-*κ*B through RhoA, p120 was knocked down by siRNA. After p120 depletion, NF-*κ*B was increased sharply (Figure 5(a)) and nuclear p65 was detected (Figure 5(b)). Although total RhoA expression was unchanged (Figure 5(a)) and p120-bound RhoA was not detected upon p120 complete knockdown (Figure 6(f)), RhoA activity was sharply upregulated (Figure 6(g)), as well as the IL-8 production (Figures 5(c) and 5(d)), compared with scrambled siRNA with and without LPS treatment.

## 4. Discussion

p120 is the prototypic member of a subfamily of Armadillo repeat domain proteins, described initially as a substrate of Src [7]. Previous studies focused on p120 regulating cell-cell adhesion, cell proliferation and polarity, embryonic development, tumor cell migration, and cancer progression [31, 32]. Recent evidence indicates that p120 appears to play an important role in suppressing inflammation in many tissues and organs [12–14]. Depletion of p120 in conditional knockout mice exhibits an inflammatory response underlying epidermis [12, 13]. p120 knockdown in a polarizing colon cancer HCA-7 cell line induces strong neutrophil attachment [15]. Consistent with observations from other tissues and experimental systems, in the present study, we have provided clear evidence that p120 downregulation in airway inflammatory response could be induced by LPS. Due to alternative splicing events in N-terminal regulatory domains, p120 has four main isoforms types 1, 2, 3, or 4 [33]. p120 isoforms 1 and 3 are mainly distributed in lung cells [34]. These two isoforms are predominantly expressed in the lung of C57 mice in our experiment. The isoform difference might be owing to the structural variations at the N-terminus. What is more, alternative splicing events also occur in the C-terminal resulting in more than four isoforms in humans [35].

NF-*κ*B participates in the control of airway epithelial inflammation processes [3, 36]. Consistent with their studies, our previous work indicated that NF-*κ*B is involved in airway epithelial inflammatory response induced by Bleomycin (BLM), and dephosphorylation of GSK3 significantly attenuated NF-*κ*B activation. Constitutively active GSK3 mutant (S9A) transfection only partially inhibits BLM-induced NF-*κ*B activation but completely inhibits I*κ*B*α* degradation [23]. We hypothesized that other mechanisms may activate NF-*κ*B signaling in airway inflammatory response.

In the present study, we found that p120 is associated with NF-*κ*B signaling. NF-*κ*B is the main regulator of inducible expression of the IL-8 gene by 5′ regulatory region gene binding sites for NF-*κ*B [37]. IL-8 is one of the principal mediators for the inflammatory response. p120 ablation in the epidermis activates an NF-*κ*B-dependent inflammatory response [12]. In our research, p120 downregulation can activate NF-*κ*B signaling and increase the IL-8 expression after LPS stimulation, which was similar to p120 knockdown. To further confirm our results, we transfected exogenous p120 isoform plasmids. However, NF-*κ*B expression was not changed in both p120 3A and p120 Δ*N* transfected groups, and the elevation of IL-8 expression after LPS treatment was significantly decreased. These results demonstrated that p120 served as an anti-inflammation factor through NF-*κ*B signaling.

But how p120 modulates NF-*κ*B activation is still unclear. The activity of RhoA is the possible intermediaries in this signaling pathway. p120 may act as a signaling nexus, conveying messages from the cellular micro- and macroenvironment to the cell's interior [38]. By regulating Rho GTPases in a context-dependent manner, p120 can exert profound effects on cellular responses [21, 38]. Initial reports revealed that p120 elicits a “dendritic-like branching” phenotype by modulating RhoA activity in fibroblasts and epithelial cells [39, 40]. In p120 knockdown epidermis, inflammation was attributed to constitutive cell autonomous activation of RhoA and downstream NF-*κ*B activity [12, 13]. Nevertheless, it is uncertain whether RhoA is involved in airway inflammation modulated by p120 through NF-*κ*B signaling.

Our study showed that although total RhoA and p120-bound RhoA were unchanged, RhoA activity is strongly increased in airway inflammation. After exogenous p120 3A and p120 Δ*N* transient transfection, RhoA activity was apparently decreased compared with MT group after LPS treatment. Moreover, p120 knockdown could significantly upregulate the activity of RhoA. Furthermore, Y27632 could partially inhibit nuclear translocation of p65. These results demonstrated that p120 modulation of NF-*κ*B signaling activation in airway inflammation is partially through RhoA. Cells with stable knockdown or overexpression of p120 may reach a new biological equilibrium to cope with an altered signal profile [24]. The changes may take some time to equilibrate, and thus RhoA activity change may not reach a detectable level after transient transfection of exogenous p120 3A and p120 Δ*N*. Nonetheless, the clear mechanism of p120 activation of RhoA is not yet known. Our study first found that RhoA directly coprecipitated with p120 in BECs. The existence of N-102-234 and N-622-628 amino acid residues of p120 may be responsible for binding with RhoA [41, 42].

In summary, our results demonstrate that p120 modulation of LPS-induced NF-*κ*B activation is partially through RhoA. p120 directly coprecipitates with RhoA; its downregulation may release RhoA and then leads to I*κ*B*α* degradation and p65 nuclear translocation, implying a possible effect of p120 on NF-*κ*B signaling in airway inflammation. RhoA may be an important mediator in p120 modulation of LPS-induced NF-*κ*B signaling. This may be useful to study the mechanism of airway inflammation and may lead to the development of novel therapeutic strategies for a number of inflammatory airway diseases.

## Abbreviations

BECs: Bronchial epithelial cells
BSA: Bovine serum albumin
ECL: Enhanced chemiluminescence
IL-8: Interleukin-8
LPS: Lipopolysaccharide
NF-*κ*B: Nuclear factor-*κ*B
p120: p120-Catenin
siRNA: Small interfering RNA.
